# Early localized alterations of the retinal inner plexiform layer in association with visual field worsening in glaucoma patients

**DOI:** 10.1371/journal.pone.0247401

**Published:** 2021-02-25

**Authors:** Rukiye Aydın, Mine Barış, Ceren Durmaz-Engin, Lama A. Al-Aswad, Dana M. Blumberg, George A. Cioffi, Jeffrey M. Liebmann, Tongalp H. Tezel, Gülgün Tezel

**Affiliations:** Department of Ophthalmology, Vagelos College of Physicians and Surgeons, Columbia University, New York, NY, United States of America; Bascom Palmer Eye Institute, UNITED STATES

## Abstract

Glaucoma is a chronic neurodegenerative disease of the optic nerve and a leading cause of irreversible blindness, worldwide. While the experimental research using animal models provides growing information about cellular and molecular processes, parallel analysis of the clinical presentation of glaucoma accelerates the translational progress towards improved understanding, treatment, and clinical testing of glaucoma. Optic nerve axon injury triggers early alterations of retinal ganglion cell (RGC) synapses with function deficits prior to manifest RGC loss in animal models of glaucoma. For testing the clinical relevance of experimental observations, this study analyzed the functional correlation of localized alterations in the inner plexiform layer (IPL), where RGCs establish synaptic connections with retinal bipolar and amacrine cells. Participants of the study included a retrospective cohort of 36 eyes with glaucoma and a control group of 18 non-glaucomatous subjects followed for two-years. The IPL was analyzed on consecutively collected macular SD-OCT scans, and functional correlations with corresponding 10–2 visual field scores were tested using generalized estimating equations (GEE) models. The GEE-estimated rate of decrease in IPL thickness (R = 0.36, P<0.001) and IPL density (R = 0.36, P<0.001), as opposed to unchanged or increased IPL thickness or density, was significantly associated with visual field worsening at corresponding analysis locations. Based on multivariate logistic regression analysis, this association was independent from the patients’ age, the baseline visual field scores, or the baseline thickness or alterations of retinal nerve fiber or RGC layers (P>0.05). These findings support early localized IPL alterations in correlation with progressing visual field defects in glaucomatous eyes. Considering the experimental data, glaucoma-related increase in IPL thickness/density might reflect dendritic remodeling, mitochondrial redistribution, and glial responses for synapse maintenance, but decreased IPL thickness/density might correspond to dendrite atrophy. The bridging of experimental data with clinical findings encourages further research along the translational path.

## Introduction

Glaucoma is a leading cause of blindness, which affects approximately 80 million people worldwide. This chronic neurodegenerative disease is characterized by the progressive loss of retinal ganglion cells (RGCs), optic nerve axons, and synapses in the retina and brain. Experimental evidence from animal models of glaucoma suggests that RGC dendrites, which are arborized in the inner plexiform layer (IPL) of the retina and establish synaptic connections with bipolar and amacrine cells, may present structural and functional alterations in earlier stages of the disease process prior to detectable loss of RGC somas. Early structural alterations in RGC dendrites, including the shrinkage and loss of dendritic branches [1–3], may lead to synaptic rearrangements [4–9]. Since synapse plasticity may allow rewiring, it has been suggested that early alterations in the RGC dendritic arbor provide a treatment window to recover synapse dysfunction and prevent further injury to RGCs in this blinding disease [10–12]. However, present observations and potential translational implications are based on animal models, and despite a few clinical studies [13–15], current understanding of IPL alterations in human glaucoma remains highly limited.

While the basic science research using animal models provides growing information about pathogenic processes of glaucomatous neurodegeneration, parallel studies aim to validate the experimental data by analysis of postmortem human tissues and analysis of the imaging-based clinical data from glaucoma patients. Undoubtedly, collaborative efforts of basic scientists and clinical researchers can speed the translation from bench-to-bedside and back for ultimate success in preventing blindness from glaucoma. In order to test the relevance of experimental observations to human glaucoma, this retrospective cohort study analyzed selected locations for glaucoma-related IPL alterations and their functional association. Morphological alterations of the IPL were analyzed using sequentially obtained macular spectral domain optical coherence tomography (SD-OCT) scans of glaucoma patients, and the functional correlation of imaging-based parameters was analyzed using visual field test results. Findings of this study that support the functional importance of early localized IPL alterations in glaucomatous eyes provide a translational path and encourage further research for improved understanding, treatment, and clinical testing of glaucoma.

## Materials and methods

### Study participants

The Institutional Review Board of Columbia University reviewed and approved this study. Informed patient consent was obtained from all participants, and the tenets of the Declaration of Helsinki were followed.

For testing the functional association of localized IPL alterations, this study enrolled a retrospective cohort of 36 patients with primary open-angle glaucoma, and a control group consisting of 18 non-glaucomatous subjects, followed for two years.

Our selection criteria required sequential high-quality SD-OCT scans (with a quality score of 7 or above, assessed by the signal strength in a 0 to 10 rating scale) obtained using the same device, and reliable visual field tests (with fixation losses ≤33%, false-positive and false-negative rates ≤15%) obtained using 10–2 standard automated perimetry (Humphrey visual field analyzer, Carl Zeiss Meditec Inc., Dublin, CA). The sequential retinal scans with approximately two-year apart (24±3 months) were obtained at high-definition (HD) 5-line raster mode with macular SD-OCT (Cirrus HD-OCT, Carl Zeiss Meditec Inc.). All scans were well-focused, well-centered and without blinking or movement artifacts or scan tilt. The mean±SD quality score of the scans was 8.74±0.99. All patients underwent 10–2 visual field testing at the same visits.

A glaucoma specialist followed all patients and their complete ophthalmic examination included the best-corrected visual acuity, the spherical equivalent refractive error, slit lamp examination, intraocular pressure measurement, central corneal thickness measurement, and dilated examination of the retina and optic disc. The same examinations were repeated at their control visits.

The glaucoma diagnosis was based on an intraocular pressure of greater than 22 mmHg by Goldmann applanation tonometry, characteristic optic disc appearance (diffuse or focal thinning of the rim), and an abnormal visual field test result obtained by 24–2 standard automated perimetry (Humphrey visual field analyzer, Carl Zeiss Meditec Inc.). All patients had open angles with no secondary causes of glaucoma. Studied patients were also required to have a best-corrected visual acuity ≥ 20/50, a refractive error between ±5.0 diopters sphere and ±3.0 diopters cylinder, and an axial length between 22 and 26 mm. Only one eye of each participant was included in the study. If both eyes of the glaucoma patients met the inclusion criteria, the eye with less advanced glaucoma was selected to allow the analysis of early alterations in the IPL.

Glaucoma patients or non-glaucomatous controls with any other ocular or neurological diseases that could affect structural or functional measurements were excluded from the study. Cataract that could affect imaging parameters and visual field sensitivities, or retinal diseases that could interfere with retinal image analysis, such as age-related macular degeneration, epiretinal membrane, or diabetic retinopathy, were also excluded. In addition, patients were excluded if they had a history of intraocular surgery or laser capsulotomy (except for uncomplicated cataract or glaucoma surgery before the initiation of study period).

### Image analysis

**Fig 1:**
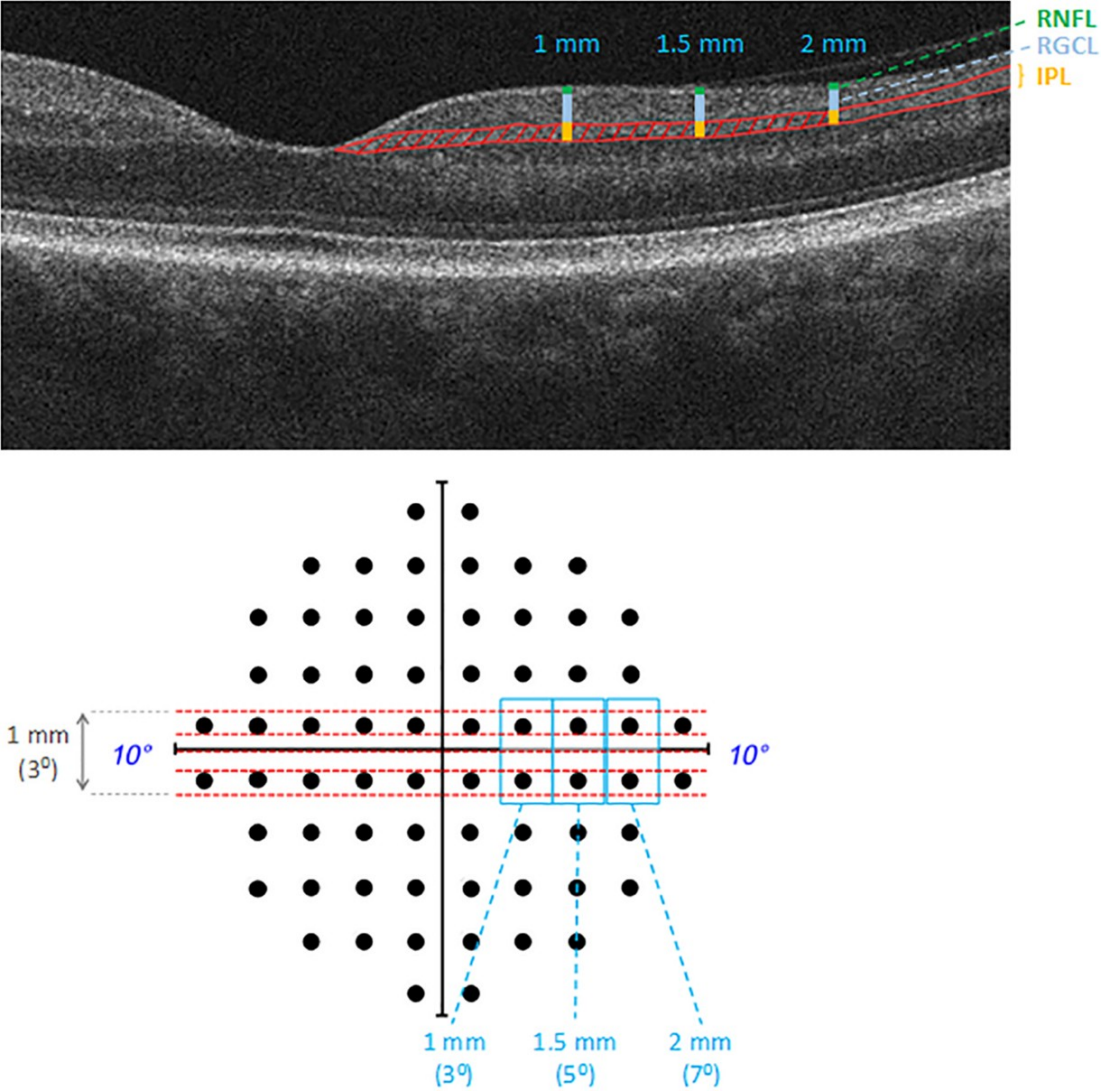
Studied imaging parameters of the IPL for testing functional associations. The imaging parameters analyzed on OCT scans included the thickness of the IPL (shown by yellow bar), RGCL (shown by blue bar), and RNFL (shown by green bar) on the nasal and temporal sides at a distance of 1, 1.5, and 2 mm from the center of the macula (shown is the nasal side). The IPL was also analyzed in the same retinal locations by linear densitometry. In addition, the IPL density was analyzed in the area within 2 mm (approximately 7^0^) from the macula center on nasal and temporal sides of the OCT scans (red patterned area). For testing the functional association of these imaging parameters, corresponding total deviation values on 10–2 visual fields were used, including one below and one above the horizontal midline within 8^0^ (shown in blue-colored boxes) on nasal and temporal fields. Red dashed lines illustrate the approximate position of 5-line raster scans that were obtained within a 6×1 mm^2^ area, the vertical span of which (1 mm) corresponds to an angular spacing of approximately 3^0^. The thickness and linear densitometry values obtained at 1, 1.5, and 2 mm from the macula center on these scans were tested against the average of total deviation scores at corresponding test points (approximately 3, 5, and 7^0^); however, the area densitometry values were tested against the average of these values.

Graphic files of high resolution horizontal 5-line raster scans (within a 6×1 mm^2^ area) were imported into image analysis program (ImageJ, National Institutes of Health, Bethesda, Maryland). The analyzed imaging parameters included the thickness of retinal nerve fiber layer (RNFL), RGC layer (RGCL), and IPL, and densitometry-based reflectance intensity of IPL as shown in **Fig 1**.

After delineating the borders of RNFL, RGCL, and IPL (using the segmented line tool of ImageJ), the local structural thickness (in μm) was analyzed and averaged on the nasal and temporal sides at a distance of 1, 1.5, and 2 mm from the center of the macula (which corresponds to an angular spacing of approximately 3, 5, and 7^0^ from the macula center).

In addition to local thickness measurements, the image analysis also included densitometry as a supplemental method to analyze alterations in the IPL-specific texture. Densitometry-based analysis included both linear densitometry and area densitometry. For the aim to supplement the analysis of association between localized structural and functional alterations, linear densitometry [16] analyzed the IPL in the same retinal locations (as described above for thickness measurements). In order to capture additional information reflecting the entire area analyzed, the IPL density was also analyzed in the area within 2 mm (approximately 7^0^) from the macula center on the nasal and temporal sides of the OCT scans (shown in **Fig 1**). At the beginning of each analysis, a scale was set to measure the thickness in μm, and the plot profile option was chosen for analysis, which gives a two-dimensional graph of reflectance intensities along a line or an area selection. The mean value that reflects the grayscale level within the area under the curve was recorded as the density value (grayscale intensity value/μm^2^).

In order to minimize imaging variability in sequentially obtained scans, we applied a normalization strategy to raw intensity data as previously described [17, 18]. The mean intensity of pixels within the IPL was therefore divided by the mean intensity of sub-IPL tissue (extending from one pixel below the posterior border of the IPL to the posterior border of the retinal pigment epithelium) and an intensity ratio (for linear and area densitometry scores, separately) was calculated for each scan. Note that the intensity scores of RNFL or RGCL were not used for normalization of the IPL grayscale intensity, because their glaucoma-related alterations are dependent on each other.

Manual image analysis was conducted by two researchers (RK and MB) masked to the patient’s other data. Repeatability and reproducibility of image analysis were tested by calculating the coefficient of variation (CoV) and the coefficient of repeatability and reproducibility (COR). In addition, the intra-class correlation coefficient (ICC) was calculated using the two-way mixed-model for absolute agreement [19]. When assessed by the CoV, the intra-examiner and inter-examiner variabilities were 1.6±0.8% (95% confidence intervals, 1.1 to 2.1) and 2.6±0.9% (95% confidence intervals, 2.0 to 3.2), respectively (the COR were 2.2±1.2% and 3.7±1.8%, respectively). The ICC for absolute agreement of the two examiners was 0.992 (95% confidence intervals, 0.971–0.998) for IPL thickness, and 0.966 (95% confidence intervals, 0.862–0.992) for IPL density. Thus, the statistical measures showed good repeatability and reproducibility of the consecutive measurements of the same scans by two examiners. In addition, all of the measurements were repeated three times, and the mean values were used for statistical comparisons.

In order to assess scan-to-scan variability, the cohort of healthy eyes was tested three times within three consecutive weeks (and then yearly over two years). The CoV for density measurements in different scans was 6.9±1.2% (95% confidence intervals, 6.35 to 7.45); while the scan-to-scan variability for thickness measurements was 1.5±0.4% (95% confidence intervals, 1.25 to 1.75). The calculated variations (which reflect the reciprocal of the signal-to-noise ratio) support our utilized strategy as a repeatable measure of reflectance intensity.

For testing the functional correlation of imaging parameters, we used corresponding total deviation values (in dB) on 10–2 visual fields (**Fig 1**). In order to minimize challenges in the perfect pairing of corresponding imaging and functional parameters, studied test points were selected with particular attention to the approximate association of perimetry test points to anatomical location, functional architecture, and receptive fields of RGCs [20–22]. As explained in **Fig 1**, by considering *(A)* the position of 10–2 visual field test points (that are located at 1^0^ from the horizontal and vertical midlines and then continue with 2^0^ intervals); and *(B)* the vertical span of 5-line raster scans (1 mm, approximately 3^0^), two test points (one below and one above the horizontal midline) were studied within 8^0^ on nasal and temporal fields. The imaging parameters, including the thickness and linear densitometry values obtained at 1, 1.5, and 2 mm from the macula center (corresponding to approximately 3, 5, and 7^0^) were tested against the average of total deviation values at corresponding two test points (at 3, 5, and 7^0^). However, the area densitometry values (that were obtained from an area within 2 mm from the macula center on temporal and nasal sides) were tested against the average of total deviation values within 8^0^ (at 3, 5, and 7^0^) on the nasal and temporal fields (excluding the values at the center of the 10–2 fields by taking the displacement of RGCs in the macula into account [22]). Thus, selected test points provide optimum proximity for an appropriate structure-function analysis.

### Statistical analysis

The statistical analysis was carried out using a statistics software, SPSS version 27 (IBM Corp., New York, NY). Group comparisons for demographic or intraocular pressure differences used the chi-square test for categorical variables and the t-test or the Mann-Whitney Rank Sum test for continuous variables. In order to account for potential association between the parameters analyzed at multiple locations in the same eye, all comparisons were performed using generalized estimating equations (GEE). The rate of change in imaging parameters and visual field scores over the follow-up was calculated by repeated-measures regression models under the GEE framework. For testing the functional association of IPL alterations, the GEE multivariate logistic model evaluated the effect of other variables on relative odds. A P value of less than 0.05 was considered statistically significant. All data are presented as mean±standard deviation accompanied by univariate or bivariate scatterplots when applicable.

## Results

Demographic and clinical characteristics of the studied patients with glaucoma and non-glaucomatous controls are presented in **Table 1**. Based on 24–2 visual field scores, the baseline stage of glaucoma was mild (31%; mean deviation, <-5.00 dB), moderate (39%; mean deviation, -5.01 to -12.00 dB), or severe (31%; mean deviation, >-12.01 dB). Throughout the two-year follow-up, the elevated intraocular pressure in glaucoma patients (>22 mmHg at diagnosis) was under control by a combination of intraocular pressure-lowering topical medications, also presented in **Table 1**.

**Table 1:** Demographic features and clinical characteristics of the study groups.

	Glaucoma	Control	Significance (P)
Number of eyes	36	18	
Age, year	68±11 (51 to 88)	66±6 (52 to 74)	0.4
Gender, female	16 (44%)	8 (44%)	1
24–2 Visual field mean deviation, dB	-9.05±10.85 (-1.65 to -26.83)	0.23±1.01 (2.06 to -1.12)	<0.001
*<-5*.*00 dB*	*11 (31%)*	*18 (100%)*	
*-5*.*01–12*.*00 dB*	*14 (39%)*	-	
*>-12*.*00 dB*	*11 (31%)*	-	
IOP, mmHg	12.83±3.09 (9 to 18)	12.78±2.69 (10 to 17)	0.9
Number of IOP-lowering medications	2.20±0.82 (1 to 3)	-	
Type of IOP-lowering medications			
*alpha-adrenergic agonists*	*15 (42%)*		
*beta-adrenergic blockers*	*14 (39%)*		
*carbonic anhydrase inhibitors*	*16 (44%)*		
*prostaglandin analogs*	*18 (50%)*		

Data are presented as mean±SD (range) when applicable.

Similar analyses were run in the group of glaucoma patients and the control group of non-glaucomatous healthy individuals over the two-year follow-up. The data from image analysis of sequential macular scans and corresponding 10–2 visual fields (mean+SD) are summarized in **Table 2**. Presented in this table include 10–2 total deviation scores (dB) and baseline thickness of RNFL, RGCL, and IPL. Besides IPL thickness, two additional sets of data from densitometry-based analysis of IPL include linear densitometry (obtained at a distance of 1, 1.5, and 2 mm from the center of the macula on nasal and temporal sides) and area densitometry values (obtained from the area within 2 mm from macula center). **Table 2** also presents the yearly progression rates of these structural and functional parameters, which were estimated by GEE. As expected, no prominent alteration was detected in non-glaucomatous controls, and their 10–2 total deviation scores remained no worse than -3.00 dB (P = 0.96) over the two-year follow-up.

**Table 2:** Summary of the data from sequential analysis of macular SD-OCT scans and visual fields.

	Baseline		Yearly Change (95% CI)	
	Control	Glaucoma	Control	Glaucoma *
Number of eyes	18	36	18	36
Retinal nerve fiber layer thickness (μm)	89.11±6.72	42.67±13.40	-0.14±0.08 (-0.30–0.20)	-0.95±0.25 (-1.43- -0.47)
Retinal ganglion cell layer thickness (μm)	91.98±5.58	47.16±13.24	-0.20±0.10 (-0.40- -0.01)	-0.94±0.14 (-1.22- -0.83)
Inner plexiform layer thickness (μm)	73.91±8.74	57.85±10.77	-0.16±0.58 (-1.11–0.79)	-1.23±0.90 (-2.99–0.54)
Inner plexiform layer linear density ratio	0.74±0.10	0.77±0.14	0.01±0.002 (0.001–0.01)	-0.03±0.01 (-0.06- -0.01)
Inner plexiform layer area density ratio	0.79±0.11	0.84±0.12	-0.004±0.08 (-0.14–0.12)	-0.04±0.08 (-0.17–0.10)
10–2 Visual field total deviation (dB)	1.18±2.12	-9.05±8.36	-0.17±0.08 (-0.32–0.02)	-0.67±0.43 (-1.51–0.16)

Data are presented as mean±SD. The rate of change over the follow-up was calculated under the generalized estimating equations (GEE) framework.

* The yearly change of the analyzed parameters were significant in the glaucoma group (P<0.001).

**Table 3** presents the baseline and follow-up IPL data in the subgroups of glaucomatous eyes divided as mild, moderate, or severe based on 24–2 visual field scores at baseline. Although the baseline thickness and density of the IPL were significantly more advanced in eyes with severe glaucoma (P<0.001), the progression of these parameters was not significantly different between subgroups (P = 0.98, and P = 0.87, respectively).

**Table 3:** IPL alterations in glaucoma subgroups with different stages.

		IPL thickness (μm)		IPL linear density ratio	
Baseline 24–2 visual field scores	Number of eyes	Baseline	Two-year follow-up	Baseline	Two-year follow-up
<-5.00 dB	11	59.98±10.38	57.30±11.98	0.80±0.11	0.71±0.17
-5.01–12.00 dB	14	59.95±9.90	58.03±14.05	0.79±0.14	0.70±0.23
>-12.00 dB	11	53.75±10.74 *	50.92±15.17	0.71±0.14 *	0.62±0.25

Data are presented as mean±SD.

* The baseline thickness and density of the inner plexiform layer (IPL) were higher in the subgroup of severe glaucoma (>-12.00 dB) than other subgroups (P<0.001); however, the alterations in IPL thickness or density over the two-year follow-up were not different between subgroups (P>0.05).

**Fig 2:**
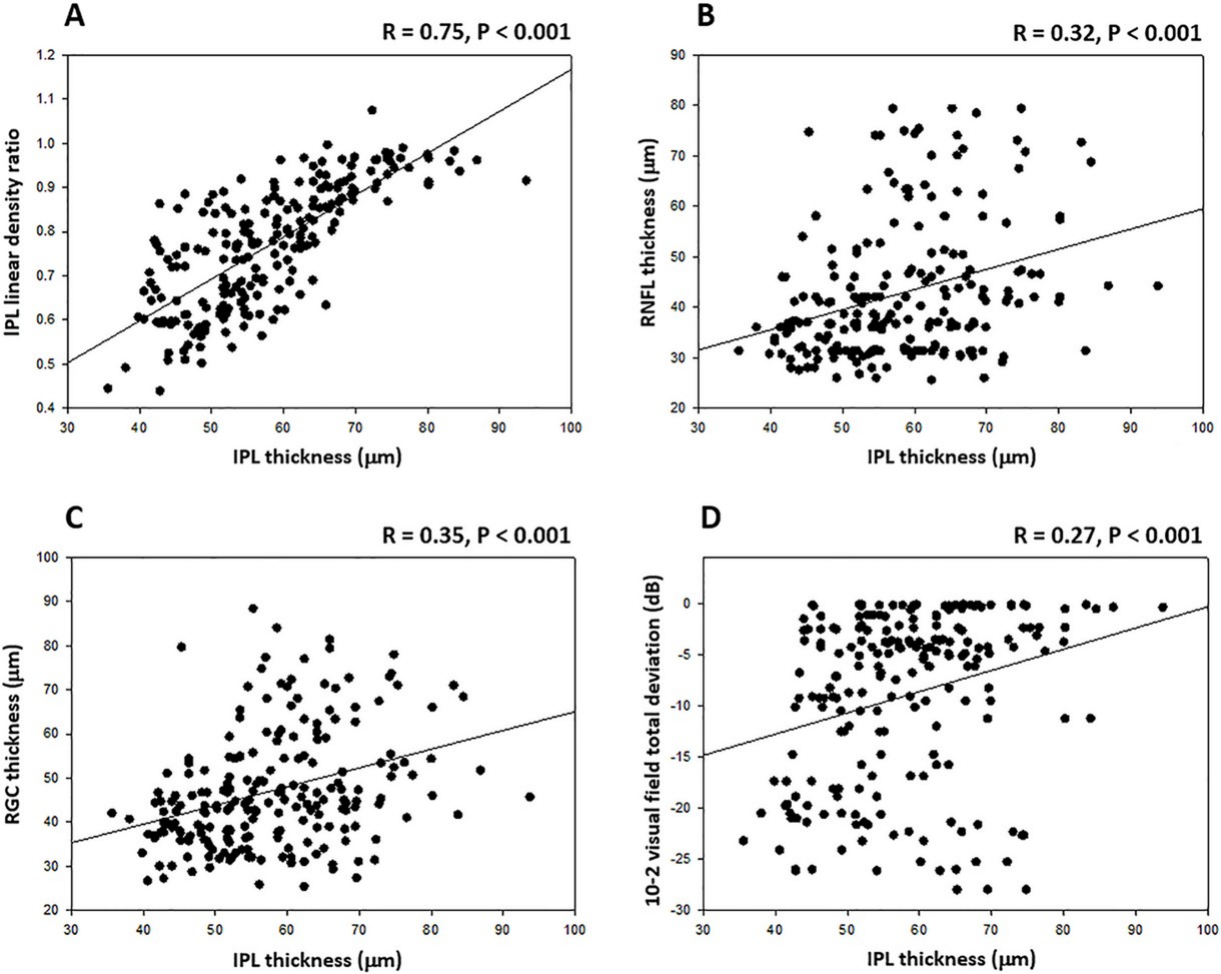
Baseline association of imaging parameters and visual field scores in patients with glaucoma. Presented include linear regression plots that show a significant relationship between the baseline values of IPL thickness and IPL linear density (**A**), IPL thickness and RNFL thickness (**B**), IPL thickness and RGCL thickness (**C**), and IPL thickness and 10–2 visual field total deviation (**D**).

Indeed, initial cross-sectional analysis of baseline parameters detected a significant correlation between RGCL and RNFL thicknesses (R = 0.92, P<0.001), RGCL thickness and visual field loss (R = 0.28, P<0.001), and RNFL thickness and visual field loss (R = 0.21, P = 0.002) in glaucomatous eyes. When analyzed cross-sectionally, the IPL thickness and density were also positively correlated (R = 0.75, P<0.001). In addition, both the IPL thickness and the linear density ratio showed a significant relationship to the stage of glaucomatous injury. As presented in **Fig 2**, the IPL thickness was significantly correlated with the RNFL thickness (R = 0.32, P<0.001), RGCL thickness (R = 0.35, P<0.001), and the total deviation scores of 10–2 visual fields (R = 0.27, P<0.001). Similarly, the IPL density presented a significant correlation with visual field scores (R = 0.89, P<0.001 for linear intensity ratios; and R = 0.78, P<0.001 for area intensity ratios). No association was detectable between the imaging parameters of IPL and the signal strength in analyzed SD-OCT scans (P>0.05).

**Fig 3:**
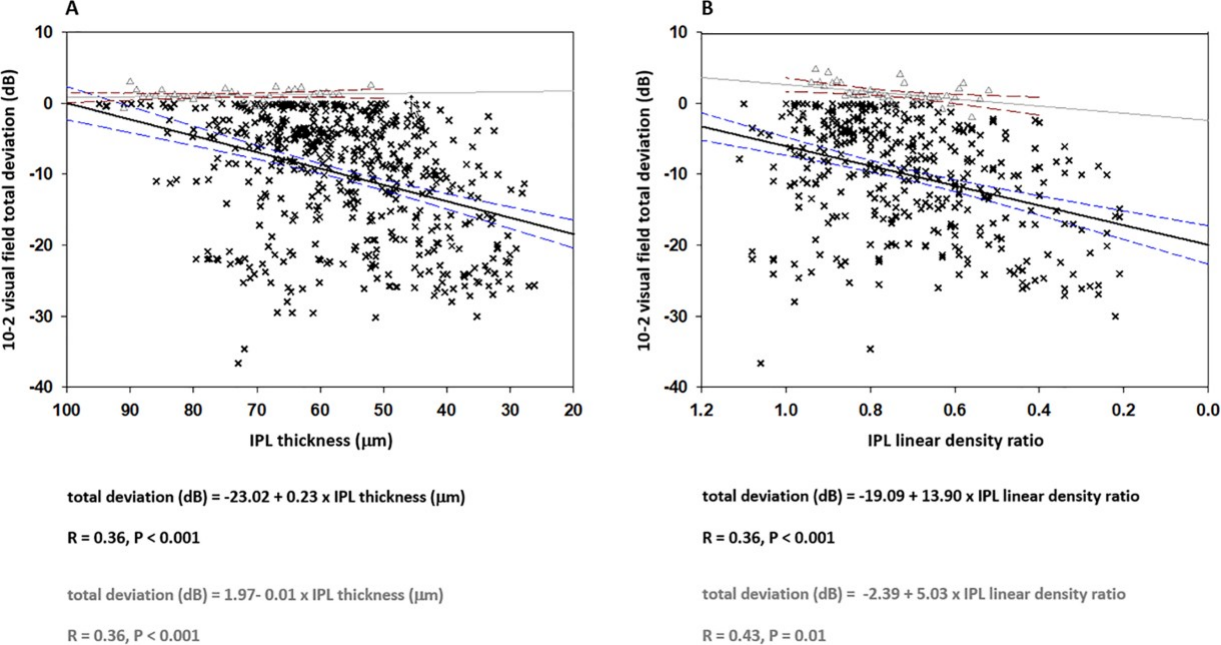
Sequential analysis of the association between IPL and visual field alterations in patients with glaucoma. Presented include the scatterplots showing a significant relationship between the alterations in IPL thickness and visual field scores (**A**), and alterations in IPL linear density and visual field scores (**B**) over the two-year follow-up. Dashed lines show the 95% confidence intervals for glaucomatous eyes (black solid line) and non-glaucomatous controls (gray solid line).

When evaluated longitudinally, IPL alterations in the glaucomatous eyes exhibited a significant correlation with alterations in visual field scores. **Fig 3** shows the IPL thickness, or IPL density, that were plotted against the visual field scores over the two-year follow-up. As presented in this figure, the alterations in IPL thickness (R = 0.36, P<0.001), or IPL density (R = 0.36, P<0.001), were significantly correlated with visual field worsening. When the rate of change in imaging and functional parameters was estimated by GEE, for every 10 μm decrease in IPL thickness per year, 10–2 visual field total deviation scores exhibited 2.3 dB decrease at corresponding locations. Similarly, the corresponding visual field scores worsened 1.39 dB for every 0.1 unit decrease in the IPL density. These associations detected between the decreasing IPL thickness (or IPL density) and the worsening of local visual field sensitivity scores were highly significant (P<0.001 for both IPL thickness and density).

However, the IPL alterations detected over the follow-up were not significantly associated with the change in RNFL thickness (R = 0.10, P = 0.11 for IPL thickness; R = 0.12, P = 0.09 for IPL density) or RGCL thickness (R = 0.12, P = 0.07 for IPL thickness; R = 0.05, P = 0.49 for IPL density) at corresponding spots in the studied glaucomatous eyes with treated intraocular pressure.

**Fig 4:**
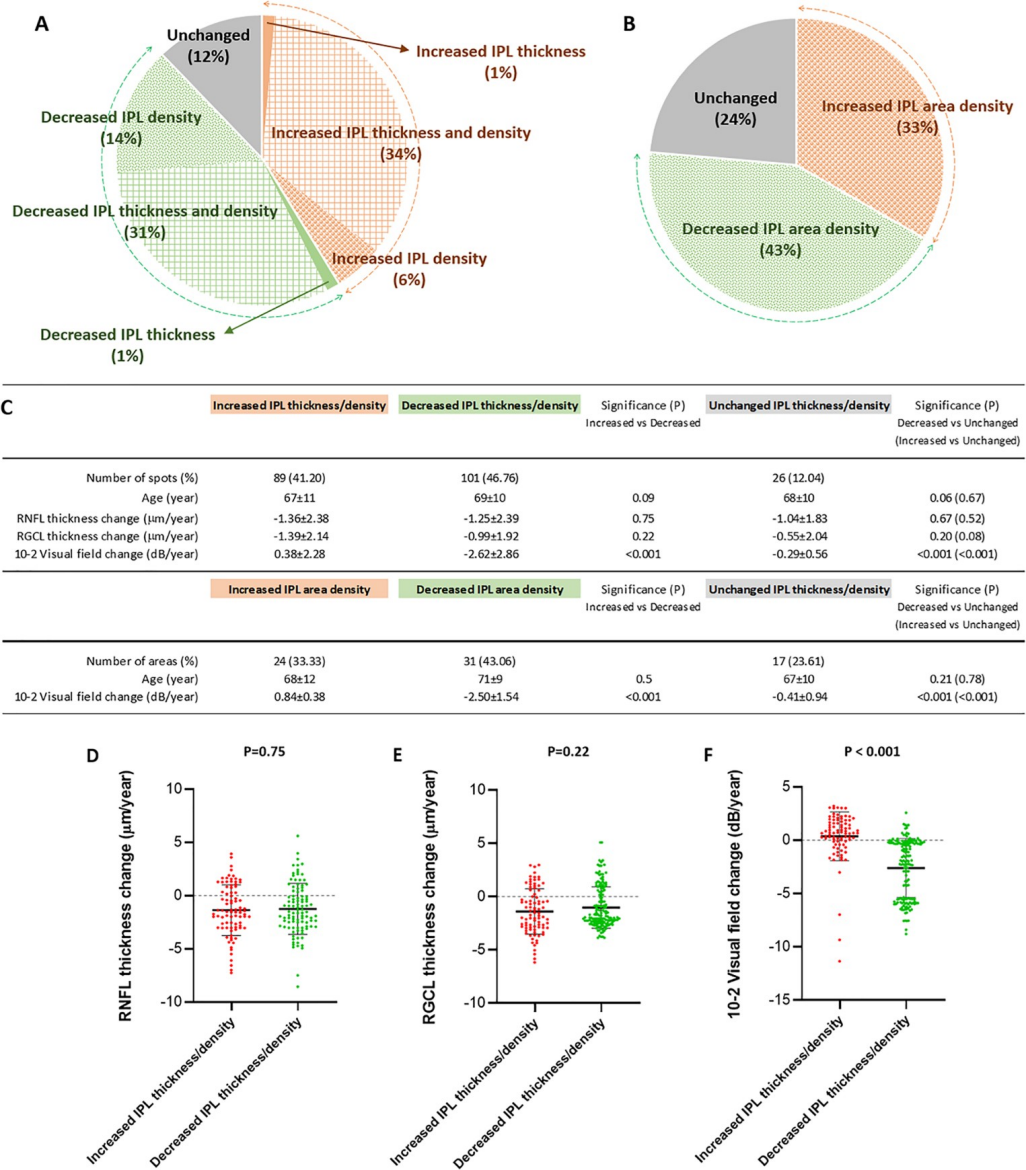
Distribution and associations of IPL alterations in patients with glaucoma. The pie graphs in panels A and B show the distribution of analysis locations with or without alterations in IPL imaging parameters over the two-year follow-up. These IPL alterations reflect those beyond the 95% confidence intervals of the change that was estimated by GEE. As shown in panel **A**, both the IPL thickness and the linear density increased at 74 (34%; red box-patterned area) analysis locations out of 216; while only the IPL thickness increased at 3 (1%; red area) or only the IPL density increased at 12 (6%; red dotted area) analysis locations. In contrast, at 67 analysis locations (31%; green box-patterned area), both the IPL thickness and the linear density decreased, while only the IPL thickness decreased at 3 (1%; green area) or only the IPL density decreased at 31 (14%; green dotted area) analysis locations. In 26 analysis locations (12%; gray area), the IPL thickness or density did not change significantly. As shown in panel **B,** the area density of the IPL (analyzed within 2 mm from the center of the macula on nasal and temporal sides) increased at 24 (33%; red area), decreased at 31 (43%, green area), or unchanged at 17 (24%; gray area) out of 72 analysis locations. Accompanying table **(C)** presents the RNFL thickness, RGCL thickness, and 10–2 visual field total deviation scores (mean±SD) for the analysis locations exhibiting increased (41%), decreased (47%), or unchanged (12%) IPL thickness and/or density, as shown in panel A. Vertical scatterplots show data distribution for RNFL thickness (**D**), RGCL thickness (**E**), and 10–2 visual field total deviation (**F**) in the subgroups with IPL alterations in different directions.

The alterations in IPL thickness, or IPL density, detected in the glaucomatous eyes were prominent at multiple analysis locations that exhibited decreased or increased values beyond the 95% confidence intervals of GEE-estimated progression rates. Comparison of the sequential image analysis data (from 216 analysis locations, including 1, 1.5, and 2 mm from the center of the macula on nasal and temporal sides) indicated that both the IPL thickness and the linear density ratio increased at 74 (34%) analysis locations out of 216; while only the IPL thickness increased at 3 (1%) or only the IPL density increased at 12 (6%) analysis locations. In contrast, at 67 analysis locations (31%), both the IPL thickness and the linear density ratio decreased, while only the IPL thickness decreased at 3 (1%) or only the IPL density decreased at 31 (14%) analysis locations. Similarly, the area density of the IPL (analyzed within 2 mm from the center of the macula on nasal and temporal sides) increased at 24 out of 72 (33%) analysis locations, or decreased at 31 (43%) analysis locations. **Fig 4** shows the distribution of analysis locations with or without IPL alterations. This figure also presents the patients’ age, imaging parameters, and visual field sensitivity scores within these clusters that exhibited IPL alterations in different directions. Comparison of these groups with decreased IPL thickness and/or density to those with increased IPL thickness and/or density showed a significant difference in progression rates of visual field scores (P<0.001). The worsening of visual field scores during sequential analyses was more significant in the group with decreasing IPL thickness/density compared to the group of increasing IPL thickness/density. However, RNFL and RGCL thicknesses were not different (P>0.05) between the two groups of IPL alterations in different directions. Similar to alterations in the thickness and linear density of IPL, alterations in the IPL area density was also associated with alterations in visual field scores. The worsening of visual field scores was more significant in locations with a decreasing area density of the IPL, as opposed to the group with an increasing area density of the IPL (P<0.001) with no age differences (P>0.05). **Fig 4** also shows vertical scatterplots for a better presentation of data distribution.

After detection of a significant association between the IPL thickness/density alterations and visual field worsening at corresponding test locations (P<0.001), additional covariates were added to the GEE model to determine for potential effects. Odds ratios were calculated for testing the impact of a set of variables (patients’ age, baseline visual field scores, and the baseline values and the concurrent rate of change in other imaging parameters). Using the GEE multivariate logistic model, the association between the IPL thickness, or IPL density, decrease and the worsening of visual field scores was independent from the patients’ age, the baseline thickness of RNFL, RGCL, or IPL, the baseline visual field scores, or the change in RNFL or RGCL thicknesses (P>0.05). **Table 4A and 4B** present the risk estimates for the functional association of IPL thickness and IPL density.

**Table 4:** 

**A. Risk estimates for the association between the IPL thickness decrease and visual field worsening**			
Parameters	**Odds Ratio**	**95% Confidence Interval**	Significance (P)
Age	1.03	0.75–1.31	0.35
Baseline RNFL thickness	0.98	0.96–1.00	0.16
Baseline RGCL thickness	0.97	0.95–0.99	0.18
Baseline IPL thickness	0.99	0.97–1.00	0.68
Baseline visual field scores	0.99	0.96–1.02	0.59
RNFL thickness change	0.95	0.93–0.96	0.32
RGCL thickness change	0.85	0.82–0.87	0.29
**B. Risk estimates for the association between the IPL density decrease and visual field worsening**			
Parameters	**Odds Ratio**	**95% Confidence Interval**	Significance (P)
Age	0.66	0.55–0.76	0.25
Baseline RNFL thickness	1.02	0.99–1.04	0.12
Baseline RGCL thickness	1.01	0.98–1.03	0.41
Baseline IPL thickness	0.99	0.96–1.02	0.66
Baseline visual field scores	1.01	0.98–1.04	0.58
RNFL thickness change	0.88	0.81–0.96	0.49
RGCL thickness change	0.63	0.52–0.74	0.48

To analyze the impacts of multiple variables on the association of IPL alterations with visual field worsening, odds ratios were calculated using the GEE multivariate logistic model. The significant association between decreasing IPL thickness/density and worsening of 10–2 visual field scores at corresponding test locations were independent from the patients’ age, the baseline thickness of RNFL, RGCL, or IPL, the baseline visual field scores, or the change in RNFL or RGCL thicknesses (P>0.05). Odds ratio is given for a 1-unit change.

**Fig 5:**
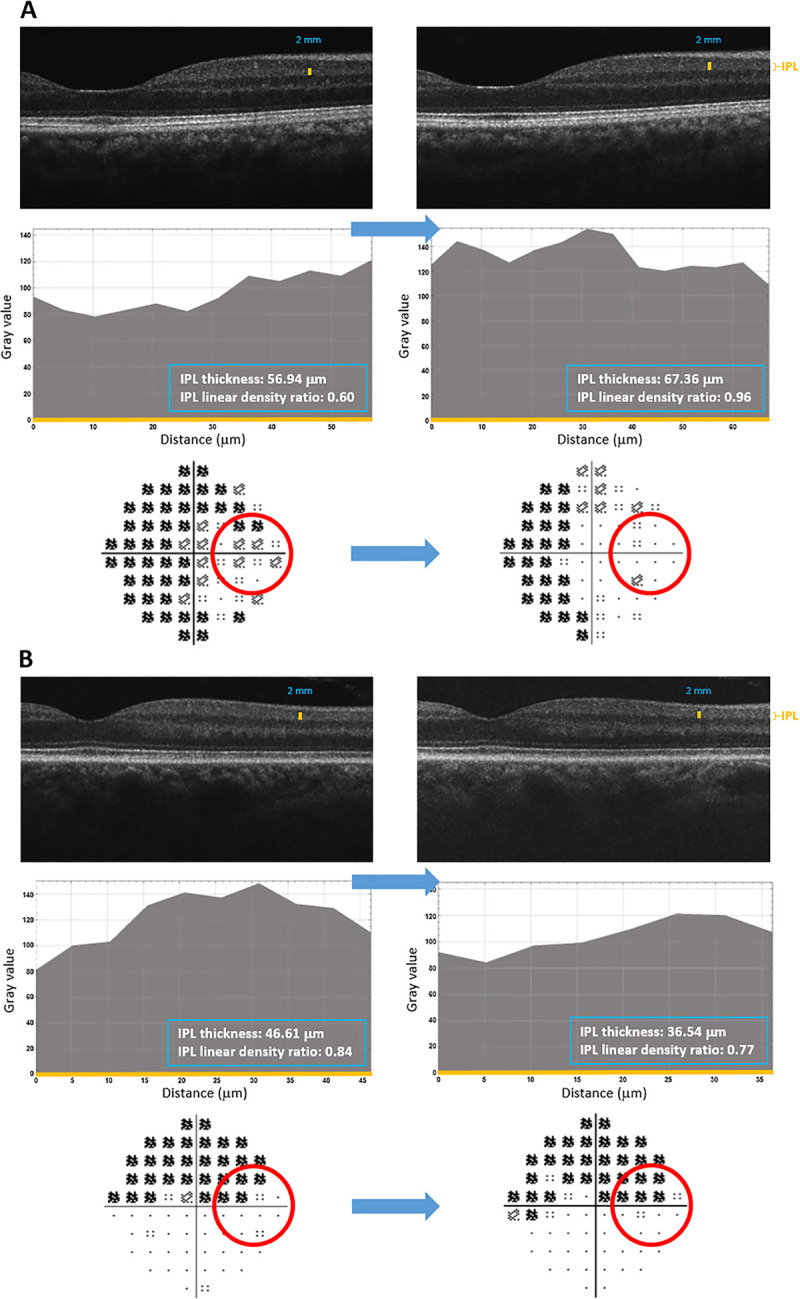
Sequential analysis of macular SD-OCT scans and 10–2 visual fields in patients with glaucoma. **A.** Temporal visual field points (red circle) corresponding to increased thickness (and density) of the IPL showed improvement. **B.** Temporal visual field points (red circle) corresponding to decreased thickness (and density) of the IPL showed worsening. The top rows in each panel show sequential macular SD-OCT scans. Middle rows in each panel show linear densitometry graphs obtained from the nasal side at 2 mm distance from the macula center. Bottom rows in each panel show 10–2 total deviation plots. The mean value of reflectance intensities that reflects the grayscale level within the area under the curve (grayscale intensity value/μm^2^) was then normalized to minimize imaging variability in sequentially obtained scans (as described in our methodology). The numbers given in the blue box are the average IPL thickness and the average IPL intensity ratio calculated after normalization. The position where these data were obtained is marked with the yellow bar on SD-OCT scans. The thickness of the IPL corresponds to the X axis of the histograms (also shown in yellow color). Note that the localized alterations of IPL, such as decreased thickness (and density), were in correlation with progressing visual field defects at corresponding locations, most of which were clustered.

**Fig 5** exemplifies localized IPL alterations and their functional correlation in sequentially analyzed glaucomatous eyes.

## Discussion

This retrospective cohort study detected localized alterations in the IPL thickness and IPL density in association with progressing local visual field defects in glaucomatous eyes. Despite a number of previous studies that analyzed the RGCL-IPL complex [23–26], studies of isolated IPL remain limited, which include a few studies that were cross-sectional and only used the IPL thickness obtained with the automated segmentation software of SD-OCT. One of those studies found that the IPL thinning is associated with RGC injury [14]. This study reported that the IPL thickness (or the thickness of the RGCL-IPL complex) shows a stronger association with corresponding 24–2 visual field scores compared with the thickness of RNFL or RGCL. However, another study found that the IPL thickness is not as good as other parameters commonly tested for glaucoma diagnosis, such as thicknesses of the isolated macular RGCL or the RGCL complex [15]. More recently, cross-sectional analysis of the macular RGCL and IPL detected no evidence for preferential IPL thinning in glaucomatous eyes, but suggested that the distance from the fovea is an important determinant of IPL thickness [27]. Although these previous studies that specifically evaluated the IPL used automated segmentation, another cross-sectional study used manual segmentation and detected a significant decrease in RGCL and IPL thicknesses in severe glaucoma [13]. Our present study analyzed the IPL in manually segmented locations in consecutive scans that were obtained approximately two years apart and found a functional association. Based on the previous studies summarized above and present findings, it appears that the analysis of IPL may complement other imaging parameters for the diagnosis and monitoring of glaucoma. This clinical aspect highly motivates additional studies to longitudinally investigate glaucoma-related IPL alterations. However, the main focus of our interest in this study remains as the clinical validation of experimental findings.

Besides alterations in retinal layer thicknesses, the potential contribution of various cellular and subcellular alterations to retinal reflectance changes is of growing interest. For example, microtubules have been shown to contribute to the reflectance of the RNFL [28]. A measurement of RNFL reflectance on SD-OCT scans has indicated glaucoma-related alterations in non-human primates [17] and humans [29, 30]. The glaucoma-related decrease in RNFL reflectance has also been found to precede the thinning of this layer in correlation with function loss in glaucomatous eyes [31, 32]. Relying on the RNFL reflectance intensity with potential to predict functional progression, the incorporation of thickness and reflectance information from SD-OCT has been suggested to improve the structure-function relation in glaucoma [18]. More recently, experimental OCT studies of the RGC5 cell line, retinal explants, and human eyes have suggested that texture measurements of the optical scatter signals derived from the subcellular network of organelles in RGCL and IPL have high potential for early clinical detection of RGC degeneration and guiding of treatment in glaucoma [33]. In fact, concentrated loss of RNFs within specific axon bundles, which leads to dark regions of RNF bundle defects, has very long been considered as one of the earliest clinical signs of glaucoma. Various other studies of glaucoma have utilized densitometry-based analysis for an evaluation of macular pigment in glaucomatous eyes [34], or an investigation of the origin of disc hemorrhages in glaucoma [35]. Notably, analysis of OCT images in age-related macular degeneration, together with high-resolution histology, has also indicated gliotic structures and mitochondria as important sources of reflectivity [36–38]. Since cellular and subcellular structures (such as mitochondria, cytoskeleton, microtubules) contribute to light scattering of the retina, our sequential image analysis in this study also included densitometry as an additional means to analyze glaucoma-related alterations in the IPL, the specific region of interest. For the aim to supplement the analysis of localized structural alterations by thickness measurements, our densitometry-based analysis included both linear densitometry (which is commonly applied in cataract studies [16] in the field of eye research, in addition to widespread applications in other systems) and area densitometry (which is commonly utilized in eye research studies). While the linear densitometry analyzed the IPL at specific locations (where layer thicknesses were measured), area densitometry aimed to capture additional information reflecting the entire area analyzed. Indeed, our reflectivity data (from linear and area densitometry) were in line with thickness measurements in correlation with the functional data.

Our findings indicated a significant relationship between localized structural alterations of the IPL (alterations in its thickness and density) and corresponding functional alterations on 10–2 visual fields (which provide higher detecting resolution than other types of perimetry [39]). As opposed to increasing thickness and/or density of the IPL, decreasing IPL thickness/density over the follow-up was found to be significantly associated with progressing visual field defects at corresponding locations (despite no prominent worsening in RNFL and RGCL thicknesses overall). One of the previous studies that analyzed the thickness of the RGCL-IPL complex in glaucoma patients similarly found a good correlation with the local loss of visual field sensitivity within approximately central 8° of fixation [21]. Another study that investigated the effect of glaucoma on different retinal layers also detected a thinner IPL in association with horizontal hemifield visual field defects [40].

It should be highlighted that our study had some limitations inherent to the retrospective study design and a relatively small group size. In order to minimize the potential sources of patient-related variability, we applied a strict eligibility criteria to select our study group of 36 eyes with primary open-angle glaucoma, in which localized imaging parameters and visual field scores were sequentially analyzed at three time points. With respect to the increasing chance of ocular and systemic health problems and the required treatments in this patient population, we considered that the inclusion of additional follow-up visits would further decrease the number of eyes fulfilling our selection criteria for this retrospective cohort study. We also thought that due to the chronic nature and asynchrony of neurodegeneration and dynamic processes of glial and healing responses in glaucoma, a two-year study period would provide clearer information about localized structure-function interaction (particularly for reflectance intensity that was utilized in addition to thickness measurements) without the cumulative addition of potential confounding impacts over a longer follow-up. Our careful set of criteria for patient selection should minimize many potential sources of sample variability and analysis error. However, the findings of this study should be further validated in prospective studies of larger and longer cohorts.

It is also important to clarify that since our study aimed to analyze the functional relevance of localized IPL alterations (but not to generate a structure-function map), selected locations on macular horizontal scans were analyzed against corresponding test points of 10–2 visual fields. However, there are common challenges of structure-function analysis, such as limitations in the perfect pairing of corresponding imaging and functional parameters, or the small stimulus size (standard size III stimulus corresponds to 0.43^0^). To minimize these problems and allow the most feasible and appropriate analysis of structural and functional parameters with optimum proximity, we carefully selected test locations. Yet, due to individual, spatial, and temporal variability, additional studies are needed for further validation and expansion of the information by testing additional locations (such as arcuate damage areas) repeatedly over a longer study period. Evolving techniques of structure-function modeling should further improve the representation of corresponding structural and functional information for such studies. Our densitometry-based analysis supplemented thickness measurements to support experimental observations of early IPL alterations with functional relevance. However, as we also discussed later below, whether intensity measurements may be useful as an additional clinical metric in the future should also be further studied.

Another important aspect to clarify relates to the challenges in assessing the glaucoma progression in imaging parameters due to measurement variability (as well as biological variability that results in individual differences in pathogenic processes). The high quality scores of the analyzed HD-OCT scans (7 or above as an inclusion criteria) that were obtained using the same device in consecutive visits, along with the good repeatability and reproducibility of manual image analysis, should minimize operator-related variability in this study. Besides avoiding off-axis fundus scanning or tilted images, analysis of macular horizontal scans (where tilting effect should be minimum), and exclusion of high myopic eyes (because myopia-related posterior segment pathology might cause position problems) should diminish the variability of retinal thickness measurements in tilted or axially stretched OCT images [41]. Concerning the imaging variability, although internal reflectivity promises useful information (and increasing resolution of SD-OCT scans makes its analysis possible), sequential comparison of pixel intensity values may be even more difficult than layer thickness measurements, because tissue reflectance varies between scans (due to differences in the exact focal plane or the quality of preretinal optics affecting light intensity). Even though we analyzed high quality scans, internal image processing (such as lossy compression of OCT scans and down sampling of graphic files) may only have minimal impact on thickness measurements (and help segmentation by clear contrast change), but may have more effect on the analysis of grayscale values. To minimize test-retest variability in pixel intensity and to better represent alterations in tissue-specific optical properties, we used a normalization strategy similar to that previously used for analysis of the RNFL reflectance intensity in glaucoma [18]. Our repeatability (as determined by the average per-eye CoV in healthy controls) was approximately 10%. The previously reported test-retest variability of the RNFL reflectance using the same measure was more than twice higher than the repeatability of IPL reflectance in our study [18]. This might be related to differences in the directionality of reflectance [42, 43] between the two retinal layers (since the RNFL contains more cylindrical structures, such as axons and their aligned cytoskeletal components), as well as equipment-related differences. We recognize that although we found a significant positive correlation between the IPL thickness and IPL density (thereby providing an internal control) and also detected a significant trend of correlation between the local alterations of IPL density and corresponding visual field scores, additional studies with methodological improvements are warranted to further verify and expand presented observations of the IPL reflectance. Our main conclusion that was based on the data derived from the multiple logistic regression analysis under the GEE framework were separately presented for the functional association of IPL thickness and IPL density changes (**Table 3**). Although alterations in IPL thickness were parallel to alterations in its density, the IPL thickness changes may be considered more robust for further discussion of our findings herein (compared to changes in density due to potential of the technical drawbacks noted).

Experimental studies of glaucoma using animal models have detected early structural alterations in RGC dendrites and synapses and accompanying function deficits prior to manifest RGC death [1–10, 44]. Neurons in the retina, similar to other brain neurons [45], have dendritic and synaptic plasticity [46, 47]. Although the number of synapses decrease as neurodegeneration progresses in ocular hypertensive animals, tissue repair and remodeling attempts are also evident prior to dendrite atrophy, synaptic pruning, and function loss. Unlike axons in the central nervous system, which are not regenerative, dendrites respond to injury, develop new dendritic branches, and expand in a larger field [48]. Experimental observations in ocular hypertensive animals similarly suggest an increase in synaptic vesicle proteins and the number of immature synapses between RGCs and bipolar cells [49].

Findings of the present study may be considered supportive of these experimental observations. A prominent alteration we detected at multiple analysis locations was increasing thickness (and density) of the IPL before further RGC and RNF loss. Interestingly, visual field sensitivity scores appeared to be more stable in test locations exhibiting increased thickness (and density) of the IPL. We wonder whether imaging-based alterations in the IPL-specific texture in the glaucomatous eyes might possibly reflect the early intrinsic recovery responses detected in experimental models, such as dendritic remodeling, accompanying mitochondrial redistribution [50], and glial responses. With respect to mitochondria being critical to energy-demanding maintenance and function of synapses [51], altered mitochondrial dynamics (such as mitochondrial transport to synapse site and/or local biogenesis in dendrites) might possibly be related to the detected increase in IPL thickness (and density). Another cellular response in the glaucomatous retina is early microglial activation as evident in human glaucoma [52] and animal models [53, 54]. Therefore, migration of microglia to the IPL (or potential alterations of Müller glia, processes of which spread through the retina and envelop synapses) might also be linked to the increased IPL thickness (and density) we detected. Evidently, these glial cells may provide early neurotrophic and metabolic/ionic/extracellular buffering support (the latter being particularly attributed to Müller glia) to stressed neurons, and may promote tissue healing by eliminating the injured dendritic structures through scavenger and phagocytosing functions [55–57]. Thus, the glaucoma-related increase in IPL thickness (and density) might possibly reflect early cellular responses for tissue repair and dendrite remodeling. The protected visual field function with increased IPL thickness (and density) may also correspond to an early and transient axogenic response to boost RGC excitability to maintain visual signaling [58]. In contrary, insufficiency or withdrawal of adaptive responses lead to dendrite atrophy that would be expected to cause tissue thinning (and density decrease) in the IPL with function loss as we detected in association with progressing visual field defects. The reduced synaptic activity drives axon degeneration, further worsening the visual function.

It is also noteworthy that the intrinsic healing attempts may reflect a longer process prior to synaptic pruning and function loss in human glaucoma that is typically treated to lower intraocular pressure, unlike animal models. However, despite an effective control of intraocular pressure in the studied eyes, sequential analysis indicated progression of visual field defects (in association with IPL alterations) in many of the test locations. Hence, intrinsic efforts or extrinsic treatments (currently limited to intraocular pressure-lowering medications) may delay, but may not always prevent, further neuron injury. With respect to synaptic plasticity and rewiring capacity, early alterations in RGC dendrites may offer a window of opportunity for further neuroprotective treatments [10]. Indeed, experimental evidence from animal models of glaucoma supports the possibility of neuroprotection to recover injured dendrites and prevent further injury to RGCs [11, 12].

In summary, the presented observations in the glaucomatous human retina may provide a clinical correlation to recent experimental findings in animal models and support a relationship between IPL alterations (that may precede other structural alterations) and progression of local visual field defects. Additional work in larger patient groups is needed to analyze IPL alterations in different regions of the retina and to assess covariate effects on structural and functional parameters more closely. In addition to patients with glaucoma, longitudinal studies of ocular hypertensive patients with no manifest glaucoma can also help collect further information about the chronology and functional association of IPL alterations. Continuously evolving imaging techniques and structure-function models are expected to accelerate the speed of progress in the field, so that increasing information should help determine whether early detection of IPL alterations in glaucomatous eyes may contribute to clinical testing of glaucoma (particularly to the emerging artificial intelligence-based diagnostic strategies). Growing knowledge gained from clinical observations should guide the interpretation of experimental findings and encourage further research towards improved treatment of this blinding disease.
